# Combined traditional Chinese medicine therapy for the treatment of infertility with polycystic ovary syndrome: A network meta-analysis of randomized controlled trials

**DOI:** 10.1097/MD.0000000000038912

**Published:** 2024-07-12

**Authors:** Yun-Peng Deng, Yan-Li Zhou, Teng-Teng Wei, Guang-Shuai He, Zhi-Xin Zhu, Shu-Ning Zhang, Mei-Jun Liu, Jing-Jing Xue, Wei-Xing Zhang, Xu-Guang Yang

**Affiliations:** a Henan University of Chinese Medicine, Zhengzhou, China; b Third Affiliated Hospital of Henan University of Chinese Medicine, Zhengzhou, China.

**Keywords:** acupuncture, Chinese medicine, infertility, network meta-analysis, polycystic ovary syndrome

## Abstract

**Background:**

Polycystic ovary syndrome (PCOS) infertility has attracted great attention from researchers due to its high incidence. Numerous studies have shown that Chinese medicine is effective in treating this disease, but there is a wide variety of Chinese medicine therapies available, and there is a lack of comparative evaluation of the efficacy of various Chinese medicine combination therapies in the clinic, which requires further in-depth exploration. This study aims to evaluate the efficacy of a combined traditional Chinese medicine (TCM) therapy for the treatment of infertility with PCOS using network meta-analysis (NMA).

**Methods:**

In PubMed, web of Science, Cochrane Library, Embase, China Knowledge Network, Wanfang Data, VIP Database, China Biomedical Literature Database (SinoMed) databases, searchs were conducted for information about the randomized controlled trials (RCTs) of combined TCM therapy for the treatment of infertility with PCOS. Quality evaluation was performed using the Cochrane 5.3 risk of bias assessment tool, and NMA using Stata 16.0.

**Results:**

This study comprised 28 RCTs using 8 combined TCM therapies in total. The results of the NMA showed that moxibustion + herbal, fire acupuncture + herbal, acupuncture + herbal, electroacupuncture + herbal, and acupoint application + herbal improved the clinical pregnancy rate better than acupuncture, herbal, and western medicines monotherapy (*P* < .05). Additionally, ear point pressure + herbal enema + herbal, acupuncture and moxibustion + herbal, fire acupuncture + herbal, and acupuncture + herbal improved the ovulation rate better than acupuncture, herbal, and western medicines monotherapy (*P* < .05). Moxibustion + herbal, fire acupuncture + herbal, and acupuncture + herbal are the 3 most effective therapies for improving the clinical pregnancy rate. Fire acupuncture + herbal, acupuncture + herbal, and ear point pressure + herbal enema + herbal are the 3 most effective therapies for improving the ovulation rate.

**Conclusion:**

The combined TCM therapy demonstrated better efficacy for the treatment of infertility with PCOS compared to acupuncture, herbal, and western medicines monotherapy. However, the optimal treatment therapy varied depending on the outcome indicators. Further large sample, high-quality, and standardized RCTs are needed to verify these findings.

## 1. Introduction

Polycystic ovary syndrome (PCOS) is a clinical condition characterized by hyperandrogenism, oligoovulation, and polycystic ovarian morphology.^[1]^ It is among the major challenges of modern society, with a complex etiology, and is a common gynecological disorder that significantly impacts the reproductive health and quality of life of women of reproductive age. The estimated prevalence of PCOS before menopause is 1 in 10 women, ranging from 5% to 15% depending on ethnicity, and affects 7% to 15% of women of reproductive age. In China, the prevalence rate is about 5.6% among women aged 19 to 45 years.^[2–5]^ Infertility due to PCOS accounts for approximately 30% of all female infertility cases and is the leading cause of anovulatory infertility.^[6,7]^ The World Health Organization predicts that infertility will be among the top 3 common diseases in the 21st century following tumors, cardiovascular, and cerebrovascular diseases, with a global prevalence rate of 8% to 12%.^[8,9]^ It has been reported^[10]^ that in China, the number of infertility patients has exceeded 50 million accounting for about 15% of women of reproductive age. The decline in female fertility due to aging will present numerous challenges to the country’s economy and society. Therefore, infertility caused by PCOS has become a significant research topic.

For the management of infertility PCOS in clinical, western drugs typically employ drugs such as letrozole, clomiphene citrate, and clomiphene. However, these drugs carry risks such as ovarian overstimulation, adverse reactions such as hot flashes, nausea, and breast pain, as well as drug resistance.^[11–13]^ Alternative, non-pharmacological treatment options such as ultrasound-guided follicular puncture and laparoscopic ovarian drilling also entail certain risks and may lead to postoperative complications like periovarian adhesions and ovarian hypoplasia.^[14]^ While assisted reproductive technologies are already in clinical use, such as intrauterine insemination and in vitro maturation culture of immature eggs are available, their efficacy remains a matter of debate; and often lead to high ovulation rate, low pregnancy rate, high miscarriage rate.^[11,15]^ In addition, ovulation induction and in vitro fertilization may increase economic burden on patients, as well as adverse effects on their psychological status.^[16–18]^ Traditional Chinese medicine (TCM) therapies such as herbal formulas, herbal extracts, and acupuncture have become increasingly important interventions for the prevention and treatment of PCOS according to extensive data analysis.^[19]^ Numerous meta-analyses have confirmed that TCM therapies are superior to western medicines and surgical treatments with regard to cycle ovulation rate, pregnancy rate, and adverse effects.^[20–26]^ Numerous clinical studies and experimental data above are conclusive in demonstrating that TCM is proficient in regulating endocrine and metabolic imbalances prevalent in PCOS with the characteristics of multi-components, multi-targets, and multi-pathways, and that the drug safety is high.^[27–32]^

However, TCM therapies are diverse and have varying efficacies. Conventional meta-analysis methods can only achieve pairwise direct comparison between single therapies. As a result, the lack of comparative evaluation of multiple TCM combination therapies hinders clinicians in clearly judging their therapeutic value. This limitation is detrimental to the exploration of the maximum advantages of TCM combination therapies and the selection of optimal treatment solutions. Network Meta-analysis (NMA), as an essential tool in evidence-based medicine, is well-suited to address this situation by calculating the comparative effectiveness of multiple therapies for a given disease, ranking them according to direct and indirect evidence, and finding the optimal therapeutic program.^[32–37]^ Therefore, this study aimed to search for randomized controlled trials of TCM treatments for PCOS infertility in recent years both domestically and internationally. We will utilize NMA to mine optimal treatment solutions for this disease. The objective of this is to provide a reliable evidence-based foundation for clinical application.

## 2. Methods

### 2.1. Registration

The protocol for this systematic review and NMA is based on the PRISMA (Preferred Reporting Items for Systematic Reviews and Meta-Analyses) protocols.^[38]^ This protocol has been registered with PROSPERO (registration number: CRD42023410217).

### 2.2. Ethics approval and consent to participate

Ethical approval and patient consent are not required since this is an overview based on published studies.

### 2.3. Inclusion criteria

#### 2.3.1. Research types

This study aims to collect all randomized controlled trials (RCTs) on the combined Chinese Medicine Protocol for infertility patients with PCOS. There are no language restrictions.

#### 2.3.2. Research participants

The enrolled patients were definitively diagnosed with infertility caused by PCOS, in compliance with the *Rotterdam criteria* which was proposed by the European Society of Reproduction and Embryology and Autonomous Sensory Meridian Response in 2003.^[39]^ Such as: ① sporadic ovulation or anovulation; ② clinical evidence of hyperandrogenemia and/or high androgen levels; ③ presence of polycystic ovaries on ultrasound (one or both ovaries having more than 12 follicles with diameters ranging from 2 to 9 mm and/or an ovary volume >10 mL). (Note: the above conditions meet 2, and exclude other diseases such as congenital adrenocortical hyperplasia, Cushing syndrome, etc). The diagnostic criteria for infertility were in line with the definitions outlined by the World Health Organization, *Obstetrics and Gynecology*,^[40]^ or *Gynecology of Traditional Chinese Medicine*,^[41]^ such as a current and uninterrupted sexual life for over a year without contraception and no conception. There were no limitations concerning the age, duration of illness, or source of the cases of the included patients.

#### 2.3.3. Interventions

*Trial group*: The combined application of TCM compounds, acupuncture, and other therapies based on TCM theory and meridian and acupoint theory is allowed, with no restrictions on the intervention start time, treatment duration, Chinese herbal selection, or acupoint selection. Control group: conventional western medicines such as Letrozole, Clomiphene, and Metformin, placebo (sham acupuncture), or one of the combined therapies (as used in the trial group).

#### 2.3.4. Outcome indicators

The following outcome indicators were considered: ① clinical pregnancy rate; ② ovulation rate.

### 2.4. Exclusion criteria

Studies that met the following criteria were excluded: ① studies with unclear or undefined diagnostic criteria. ② Duplicate studies published in multiple languages or formats, such as journals and conferences. ③ Studies that include patients with infertility not solely attributed to PCOS or those with multiple pathologies. ④ Non-RCT trials, animal studies, reviews, systematic evaluations, and meta-analysis. ⑤ Studies that lack complete data and cannot be remedied by contacting the authors. ⑥ Studies that demonstrate noncompliance with intervention. ⑦ Studies that lack endpoint indicators related to infertility with PCOS.

### 2.5. Retrieval strategy

The search strategy adopted a combination of subject and free words. We will use computers to search 8 databases, including PubMed, Cochrane Library, Web of Science, Embase, China Knowledge Network, Wanfang Database, VIP Database, and Chinese Biomedical Literature Database (SinoMed) from build database until February 28, 2023. Subject terms and free terms were utilized in a joint search, based on the characteristics of each database, and the search formula was developed and adjusted accordingly. The search terms include: “acupuncture,” “Traditional Chinese Medicine,” “polycystic ovarian syndrome,” “infertility,” et al Taking PubMed as an example, the search strategy is provided in Table 1.

**Table 1:** PubMed database search strategy.

#1	((((“Medicine, Chinese Traditional”[Mesh]) OR (“Acupuncture Therapy”[Mesh])) OR (((((((((((((Chung I Hsueh[Ti/Ab]) OR (Hsueh, Chung I[Ti/Ab])) OR (Traditional Medicine, Chinese[Ti/Ab])) OR (Zhong Yi Xue[Ti/Ab])) OR (Chinese Traditional Medicine[Ti/Ab])) OR (Chinese Medicine, Traditional[Ti/Ab])) OR (Traditional Tongue Diagnosis[Ti/Ab])) OR (Tongue Diagnoses, Traditional[Ti/Ab])) OR (Tongue Diagnosis, Traditional[Ti/Ab])) OR (Traditional Tongue Diagnoses[Ti/Ab])) OR (Traditional Tongue Assessment[Ti/Ab])) OR (Tongue Assessment, Traditional[Ti/Ab])) OR (Traditional Tongue Assessments[Ti/Ab]))) OR ((((((((((((((((((((((((Acupuncture Treatment[Ti/Ab]) OR (Acupuncture Treatments[Ti/Ab])) OR (Treatment, Acupuncture[Ti/Ab])) OR (Therapy, Acupuncture[Ti/Ab])) OR (Pharmacoacupuncture Treatment[Ti/Ab])) OR (Treatment, Pharmacoacupuncture[Ti/Ab])) OR (Pharmacoacupuncture Therapy[Ti/Ab])) OR (Therapy, Pharmacoacupuncture[Ti/Ab])) OR (Acupotomy[Ti/Ab])) OR (Acupotomies[Ti/Ab])) OR (Chinese medicine[Ti/Ab])) OR (TCM[Ti/Ab])) OR (Acupuncture[Ti/Ab])) OR (warming-needle moxibustion[Ti/Ab])) OR (electro-acupuncture[Ti/Ab])) OR (Electroacupuncture[Ti/Ab])) OR (point injection therapy[Ti/Ab])) OR (water injection[Ti/Ab])) OR (point application therapy[Ti/Ab])) OR (catgut embedment in acupoint[Ti/Ab])) OR (red-hot needling[Ti/Ab])) OR (fire needling[Ti/Ab])) OR (ear point[Ti/Ab])) OR (auricular acupoint[Ti/Ab]))) OR (“Moxibustion”[Mesh])
#2	(“Polycystic Ovary Syndrome”[Mesh]) OR ((((((((((((((Ovary Syndrome, Polycystic[Ti/Ab]) OR (Syndrome, Polycystic Ovary[Ti/Ab])) OR (Stein-Leventhal Syndrome[Ti/Ab])) OR (Stein Leventhal Syndrome[Ti/Ab])) OR (Syndrome, Stein-Leventhal[Ti/Ab])) OR (Sclerocystic Ovarian Degeneration[Ti/Ab])) OR (Ovarian Degeneration, Sclerocystic[Ti/Ab])) OR (Sclerocystic Ovary Syndrome[Ti/Ab])) OR (Polycystic Ovarian Syndrome[Ti/Ab])) OR (Ovarian Syndrome, Polycystic[Ti/Ab])) OR (Polycystic Ovary Syndrome 1[Ti/Ab])) OR (Sclerocystic Ovaries[Ti/Ab])) OR (Ovary, Sclerocystic[Ti/Ab])) OR (Sclerocystic Ovary[Ti/Ab]))
#3	(“Infertility, Female”[Mesh]) OR (((((((((((Female infertility[Ti/Ab]) OR (Female Infertility[Ti/Ab])) OR (Sterility, Postpartum[Ti/Ab])) OR (Postpartum Sterility[Ti/Ab])) OR (Subfertility, Female[Ti/Ab])) OR (Female Subfertility[Ti/Ab])) OR (Sub-Fertility, Female[Ti/Ab])) OR (Female Sub-Fertility[Ti/Ab])) OR (Sub Fertility, Female[Ti/Ab])) OR (Sterility, Female[Ti/Ab])) OR (Female Sterility[Ti/Ab]))
#4	“randomized controlled trial”[Mesh] OR “RCT”[Mesh] OR “randomly”[Ti/Ab] OR “randomized”[Ti/Ab] OR “controlled clinical trial”[Ti/Ab] OR “clinical trial”[Ti/Ab]
#5	#1 AND #2 AND #3 AND #4

### 2.6. Literatures screening and data collection

Two researchers independently conducted a thorough literature review, extracted pertinent information, cross-checked for accuracy, and established a comprehensive database. Disagreements regarding the literature were resolved through consultation with other qualified researchers. EndNote (V.X.9.2) software was used for screening literatures: ① delete duplicate literature; ② read the title and abstract to exclude literature; ③ read the full text for secondary screening according to inclusion and exclusion criteria. (Full text information could be obtained by e-mail and other means of authors if needed during the screening process.)

Data collection: ① title, author, publication time, etc; ② baseline characteristics and interventions; ③ elementary risk of bias evaluation indicators; ④ outcome indicators measurements data.

### 2.7. Assessment of the risk of bias

Risk of bias was evaluated according to the quality evaluation tool (RoB 2.0) recommended in the Cochrane Handbook 5.1.0.^[42]^ The assessment was conducted by 2 investigators independently, cross-checked, and any discrepancies were resolved through discussion with the other investigators. Evaluation contents: random sequence generation, allocation concealment, blinding, incomplete outcome data, selective outcome reporting, and other possible biases. According to the criteria, each item was judged as “low risk,” “high risk,” or “unclear risk.”

### 2.8. Statistical analysis

The outcome indicators in this study were all dichotomous variables, and utilized odds risk (OR) as the effect size, and its 95% confidence interval (CI) was calculated. Taking into account inter-study heterogeneity, the data were analyzed using a random effects model.^[43]^ NMA was conducted using Stata 16.0 software by using the network package command. The evidence network diagram depicts the number of patients who received the intervention, with larger dots indicating more significant numbers, while the thickness of the line between 2 interventions represents the number of studies included.^[44]^ The area under curve of surface under the cumulative ranking (SUCRA) was expressed as a percentage (range, 0–100%) to explain the superiority or inferiority of each intervention, and the percentage size is proportional to the efficacy of the intervention.^[45–47]^ In addition, a “comparison-corrected” funnel plot was used to assess the publication bias and small sample of included studies.^[48]^

## 3. Results

### 3.1. Results of study identification and selection

**Figure 1.**
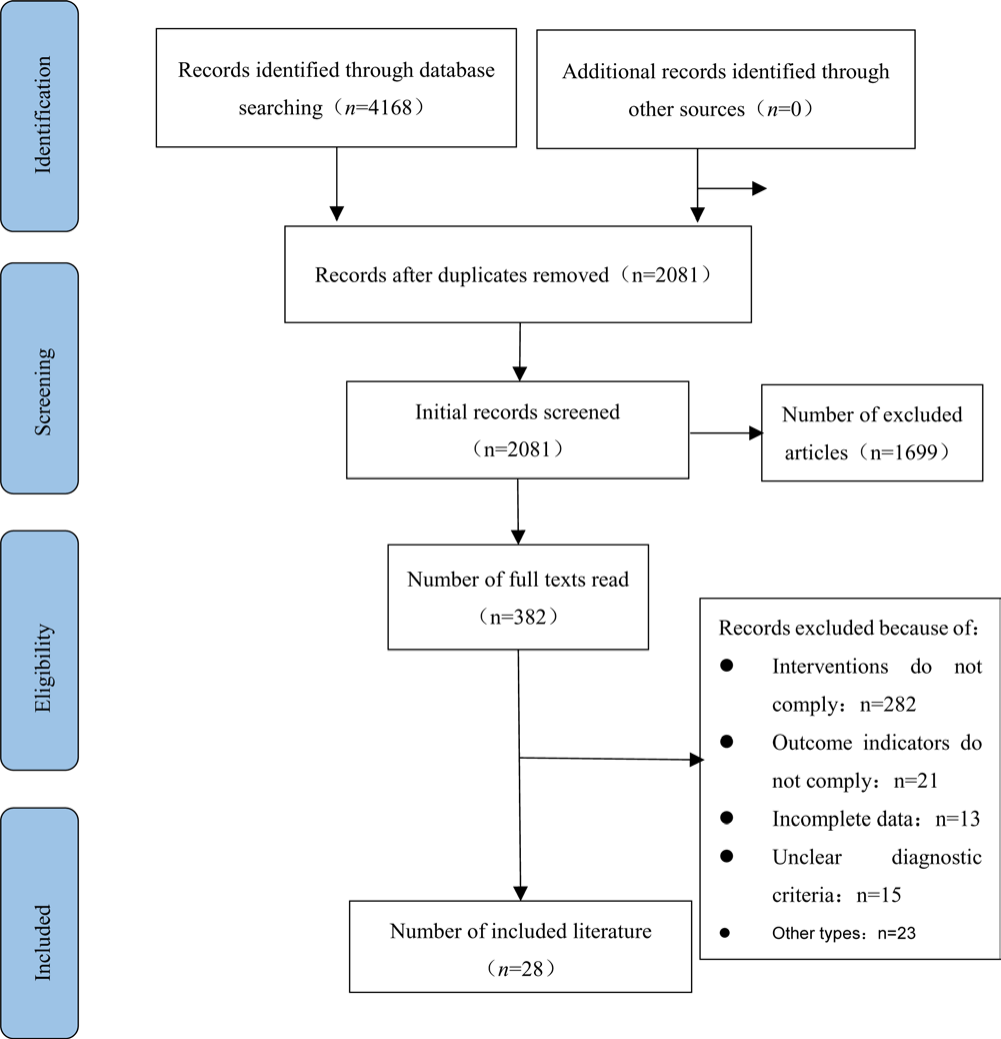
Literature search process.

Through a rigorous search of diverse databases, a total of 4168 articles were initially identified. This consisted of 978 articles sourced from China Knowledge Network, 875 articles retrieved from Wanfang Database, 661 articles obtained from VIP Database, 1020 articles obtained from SinoMed, 57 articles retrieved from Cochrane Library, 293 articles obtained from Embase, 52 articles obtained from PubMed, and 232 articles obtained from Web of Science. Following meticulous evaluation of the title and abstract, 1699 articles were excluded, leaving only 28 RCTs^[49–76]^ for further assessment and meta-analysis. The screening process and results for this stage are depicted in Figure 1.

### 3.2. Characteristics of included studies

We finally included 28 RCTs were published in Chinese, except for 3^[61–63]^ three-arm experiments, all double-arm experiments with a total of 2771 participants, 1300 of whom were assigned to the intervention group and 1471 to the control group. The publication time of the studies was distributed from 2012 to 2022. There were 11 different interventions were involved, including moxibustion + herbal,^[49–54]^ electro-acupuncture + herbal,^[55]^ ear point pressure + herbal enema + herbal,^[56]^ fire needling + herbal,^[57]^ catgut embedment in acupoint + herbal,^[58]^ acupoint application + herbal,^[59]^ acupuncture + herbal,^[60–64,70–76]^ acupuncture and moxibustion + herbal,^[65–69]^ western medicine,^[49–55,58,65,70–76]^ herbal,^[56,57,59,61–64,66–69]^ and acupuncture.^[60–63]^ The outcome of the included studies was the pregnancy rate or the ovulation rate. The basic information of the included studies is shown in Table 2.

**Table 2:** List of basic features included in the study.

Study	Test group				Control group				Treatment (menstrual cycle)	Outcome measure
	Intervention	Cases	Age (years)	Course of disease(years)	Intervention	Cases	Age (years)	Course of disease(years)		
Jia 2012^[49]^	MH	60	28.35 ± 4.16	3.89 ± 1.76	W	60	24.49 ± 4.02	3.79 ± 1.93	3	①②
Xu 2014^[50]^	MH	128	28.27 ± 4.56	3.72 ± 1.59	W	122	28.41 ± 5.02	3.78 ± 1.63	3	②
Zhu 2019^[51]^	MH	30	26.24 ± 5.20	3.48 ± 1.63	W	30	26.58 ± 4.82	3.35 ± 1.72	3	①②
Si 2016^[52]^	MH	43	27.90 ± 5.00	4.00 ± 2.10	W	43	28.40 ± 4.80	3.70 ± 2.00	3	②
Lu 2019^[53]^	MH	50	28.40 ± 3.70	4.40 ± 0.70	W	50	28.80 ± 3.90	4.20 ± 0.80	3	①②
Liu 2107^[54]^	MH	43	59.70 ± 12.80	–	W	43	59.70 ± 12.80	–	6	②
Peng 2018^[55]^	EAH	60	26.41 ± 2.54	–	W	60	25.14 ± 3.85	–	3	②
Zhu 2016^[56]^	EHH	30	27.35 ± 3.26	4.10 ± 1.50	H	28	28.38 ± 4.19	4.20 ± 2.10	3	①②
Geng 2020^[57]^	FH	67	31.00 ± 7.00	3.24 ± 0.74	H	67	31.00 ± 7.00	3.22 ± 0.76	40 days	①②
Zhu 2020^[58]^	CH	30	26.37 ± 5.31	3.63 ± 1.58	W	30	26.47 ± 5.62	3.41 ± 1.49	3	①②
Shi 2022^[59]^	AAH	44	29.56 ± 1.09	–	H	44	29.36 ± 1.12	–	3months	②
Zhou 2022^[60]^	AH	40	26.87 ± 2.19	3.15 ± 0.48	A	40	27.13 ± 2.26	2.82 ± 0.37	3	①②
Ren 2022^[61]^	AH	80	28.00 ± 5.00	4.03 ± 0.59	A	80	29.00 ± 5.00	4.05 ± 0.61	3 months	①②
					H	80	17.00 ± 5.00	3.95 ± 0.62	3 months	①②
Lei 2021^[62]^	AH	70	31.00 ± 4.00	3.50 ± 0.90	A	70	32.00 ± 5.00	3.50 ± 0.6	3	①②
					H	70	31.00 ± 4.00	3.40 ± 0.70	3	①②
Yang 2022^[63]^	AH	30	30.20 ± 3.21	3.40 ± 0.35	A	30	29.90 ± 3.05	3.40 ± 0.37	3	①②
					H	30	29.8.0 ± 2.87	3.30 ± 0.29	3	①②
He 2021^[64]^	AH	33	25.57 ± 0.75	1.64 ± 0.03	H	33	25.72 ± 0.73	1.64 ± 0.03	3	②
Jiang 2015^[65]^	AMH	40	29.00 ± 3.00	3.80 ± 2.10	W	40	28.00 ± 3.00	4.10 ± 1.90	3	②
Zhong 2019^[66]^	AMH	63	33.00 ± 5.00	1.50 ± 0.62	H	63	33.00 ± 5.00	1.53 ± 0.60	6	①②
Peng 2022^[67]^	AMH	32	29.91 ± 4.54	–	H	32	28.47 ± 3.10	–	–	①②
Cui 2015^[68]^	AMH	33	31.38 ± 4.87	1.54 ± 1.01	H	33	33.45 ± 3.75	1.43 ± 0.79	6	②
Qiao 2012^[69]^	AMH	30	–	–	H	30	–	–	6	①②
Wang 2013^[70]^	AH	28	30.30 ± 2.70	7.00 ± 2.70	W	28	31.00 ± 3.10	7.00 ± 2.90	3	②
Shuai 2017^[71]^	AH	33	28.59 ± 1.57	2.55 ± 1.82	W	32	29.21 ± 1.97	2.32 ± 1.51	3 months	①②
Luo 2018^[72]^	AH	30	29.63 ± 2.69	3.87 ± 1.73	W	30	30.21 ± 3.19	3.48 ± 1.77	3	②
Li 2021^[73]^	AH	33	29.63 ± 4.02	5.38 ± 1.24	W	33	29.53 ± 4.09	5.39 ± 1.22	3	①②
Yu 2020^[74]^	AH	55	26.56 ± 6.11	2.38 ± 0.89	W	55	25.39 ± 4.89	3.59 ± 0.75	2–6	②
Gao 2017^[75]^	AH	45	28.89 ± 2.50	2.81 ± 1.30	W	45	29.05 ± 2.32	2.98 ± 1.26	–	①②
Li 2019^[76]^	AH	40	33.10 ± 1.60	4.30 ± 0.50	W	40	32.80 ± 1.70	4.10 ± 0.70	3 months	①②

*Notes*: –: not reported, MH: moxibustion + herbal, EAH: electro-acupuncture + herbal, EHH: ear point pressure + herbal enema + herbal, FH: fire needling + herbal, CH: catgut embedment in acupoint + herbal, AAH: acupoint application + herbal, AH: acupuncture + herbal, AMH: acupuncture and moxibustion + herbal, W: western medicine, H: herbal, A: acupuncture, ①: pregnancy rate, ②: ovulation rate.

### 3.3. Quality assessment of the included studies

**Figure 2.**
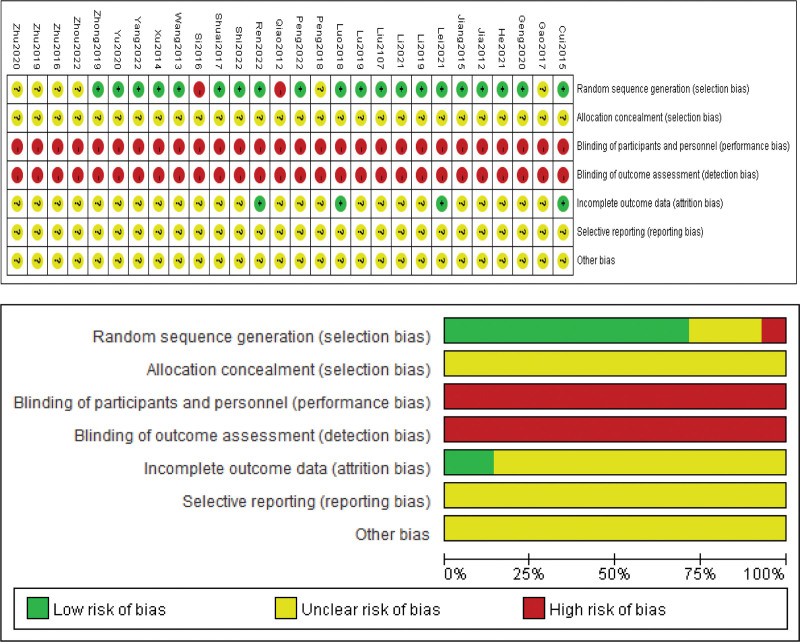
Literature quality evaluation results.

We assessed the risk of bias for each study using the Cochrane RoB–2.0 tool. The generation method of specific random sequences was reported by 20 studies, of which 17 studies^[49,50,53,54,57,59,61–66,68,70,72–74]^ used random number table for random allocation, one study^[76]^ used lottery for random allocation, rated as “low risk.” One study^[69]^ used sequence of medical visits, one study^[52]^ used treatment methods, both in non-randomized grouping, rated as “high risk.” Meanwhile, 8 studies^[51,55,56,58,60,65,67,71]^ mentioned randomized grouping, but failed to specify the specific way of generating the randomized sequence, rated as “unclear risk.” None of all studies^[49–76]^ mentioned whether allocation concealment was performed was evaluated as “unclear risk.” None of all studies^[49–76]^ mentioned the use of blinding for patients, intervention implementers, data analysts, and outcome assessors, which was evaluated as “high risk.” In 4 studies^[61,62,68,72]^ detailed information concerning shedding rates and reasons for missing cases were reported, the missing data were insufficient to affect the predicted intervention effect therefore classified as “low risk”; and the remaining were rated as “unclear risk.” None of all studies^[49–76]^ mentioned clinical study registration information, and the complete study protocols were not available hence classified as “unclear risk.” There was no mention of other biases within any of the studies,^[49–76]^ which were categorized as “unclear risk.” The evaluation table for risk of bias is depicted in Figure 2.

### 3.4. Evidence network

**Figure 3.**
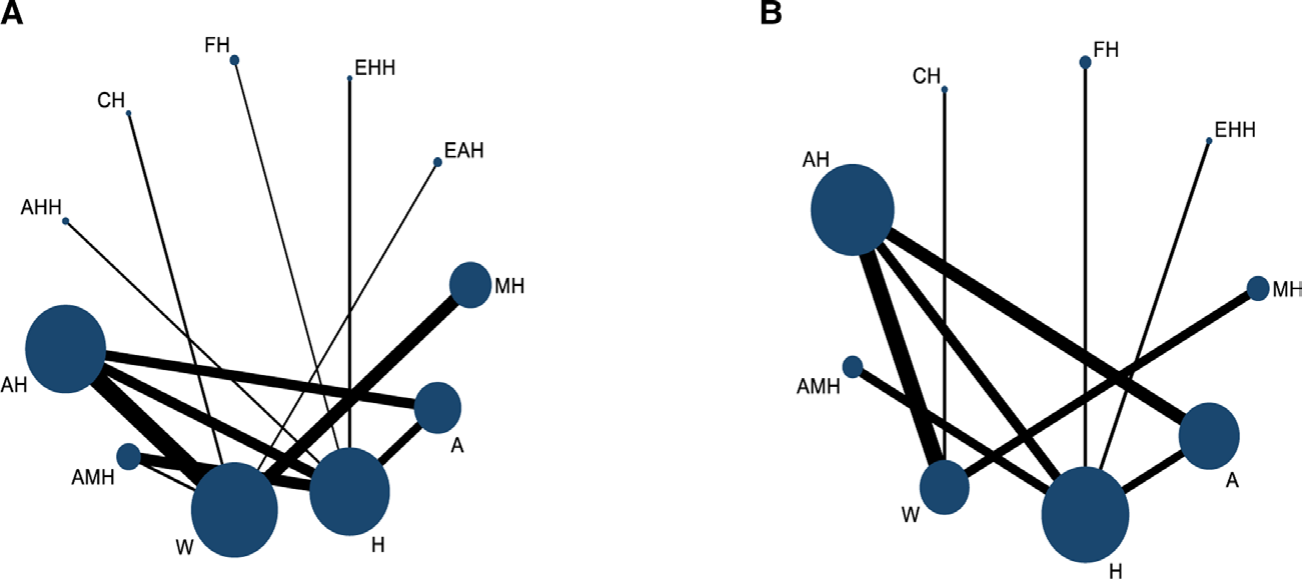
Evidence network relationship figure.

The reticulation diagram depicts individual interventions through the use of dots, the size of each dot corresponds to the sample size of the included cases, and the thickness of the lines signifies the sample size of the studies analyzed for inclusion in the analysis. Additionally, 2 connected dots indicate a direct comparison between the interventions, while unconnected dots represent indirectly analyzed comparisons through NMA. All the studies^[49–76]^ presented data on pregnancy rates, which included 11 different interventions and the network relationship was centered on western medicine therapy (Fig. 3A). In addition, 18 studies^[51,53,56–58,60–63,65–67,69,71,75,76]^ reported the ovulation rate, involving 9 interventions and the network relationship was centered on herbal therapy (Fig. 3B).

### 3.5. Network meta-analysis

#### 3.5.1. Pregnancy rate

**Figure 4.**
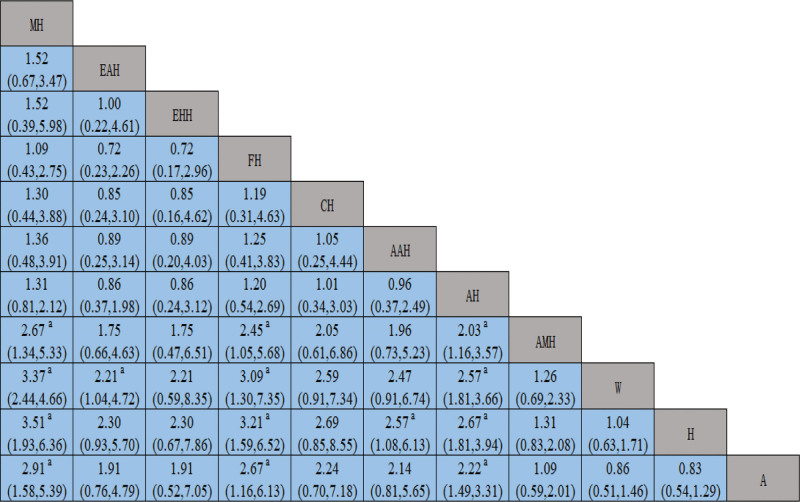
Network meta-analysis of the pregnancy rate.

The NMA of the yielded studies resulted in 55 pairwise comparisons. By combining the OR and 95% CI, the NMA results revealed that MH, FH, AH had better intervention effects compared to AMH, A, H, and W. Additionally, EAH showed better efficacy than W, and AAH showed better efficacy than H. All of the above differences were statistically significant (*P* < .05). No statistically significant differences in the comparisons (*P* > .05) were observed between the other interventions, as demonstrated in Figure 4.

#### 3.5.2. Ovulation rate

**Figure 5.**
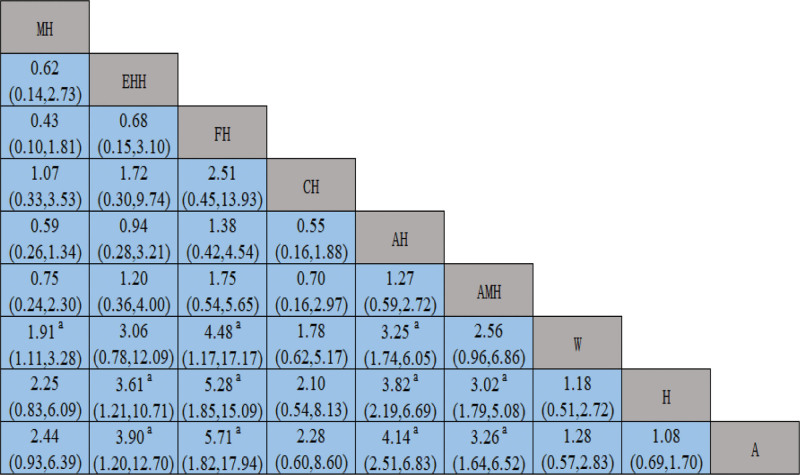
Network meta-analysis of the ovulation rate.

The NMA of the included studies yielded 36 pairwise comparisons. Combining the OR and 95% CI, the results of NMA showed that MH had better efficacy than W and EHH; compared with W, H, the intervention effect of EHH, and AMH was better. Furthermore, FH and AH showed better efficacy than W, H, and A. All of the above differences were statistically significant (*P* < .05). Conversely, there were no statistically significant differences in the comparisons (*P* > .05) between the other interventions, as illustrated in Figure 5.

### 3.6. SUCRA probability ranking

#### 3.6.1. Pregnancy rate

**Figure 6.**
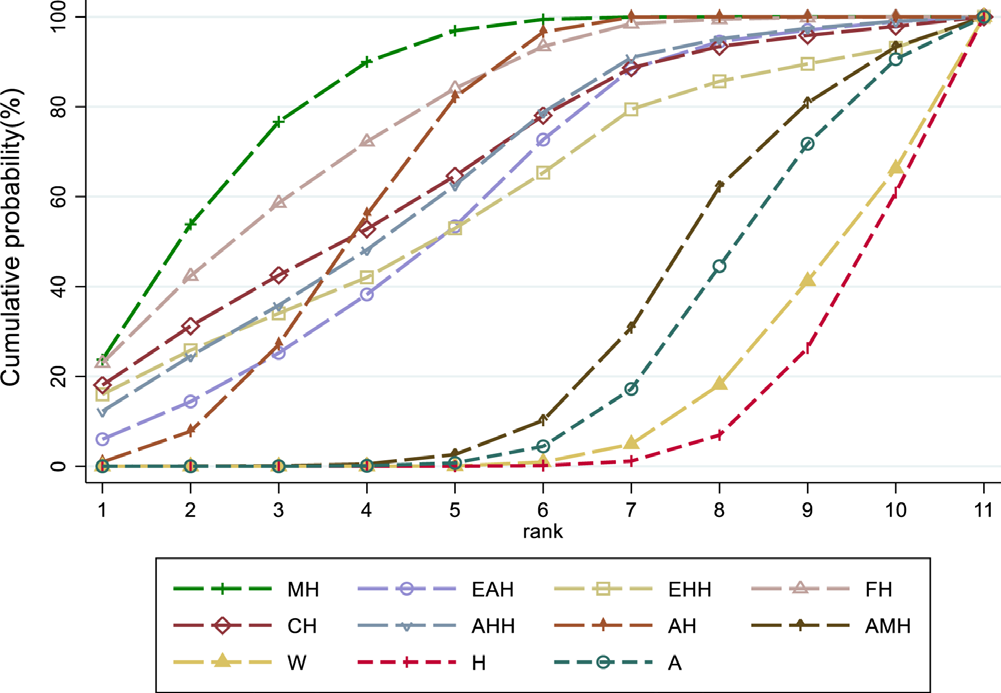
SUCEA of the pregnancy rate.

According to SUCRA results, MH appears to be the most efficacious intervention. The SUCRA probability ranking, from highest to lowest, is as follows: MH (84.1%) > FH (77.1%) > AH (67.0%) > CH (66.3%) > AAH (64.5%) > EAH (58.9%) > EHH (58.4%) > AMH (28.1%) > A (22.9%) > W (13.1%) > H (9.5%), as depicted in Figure 6.

#### 3.6.2. Ovulation rate

**Figure 7.**
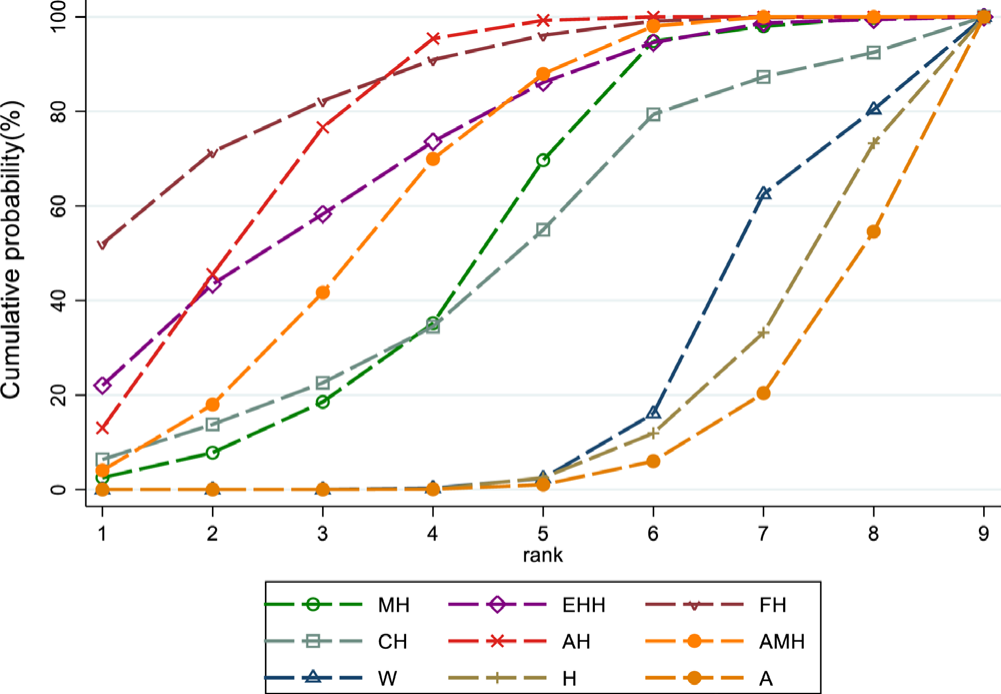
SUCRA of the ovulation rate. SUCRA = surface under the cumulative ranking.

According to SUCRA results, FH appears to be the most effective intervention, with the SUCRA probability ranking highest to lowest as follows: FH (86.5%) > AH (78.7%) > EEH (72.0%) > AMH (65.0%) > MH (53.3%) > CH (48.9%) > W (20.2%) > H (15.1%) > A (10.3%), as shown in Figure 7.

### 3.7. Publication bias

**Figure 8.**
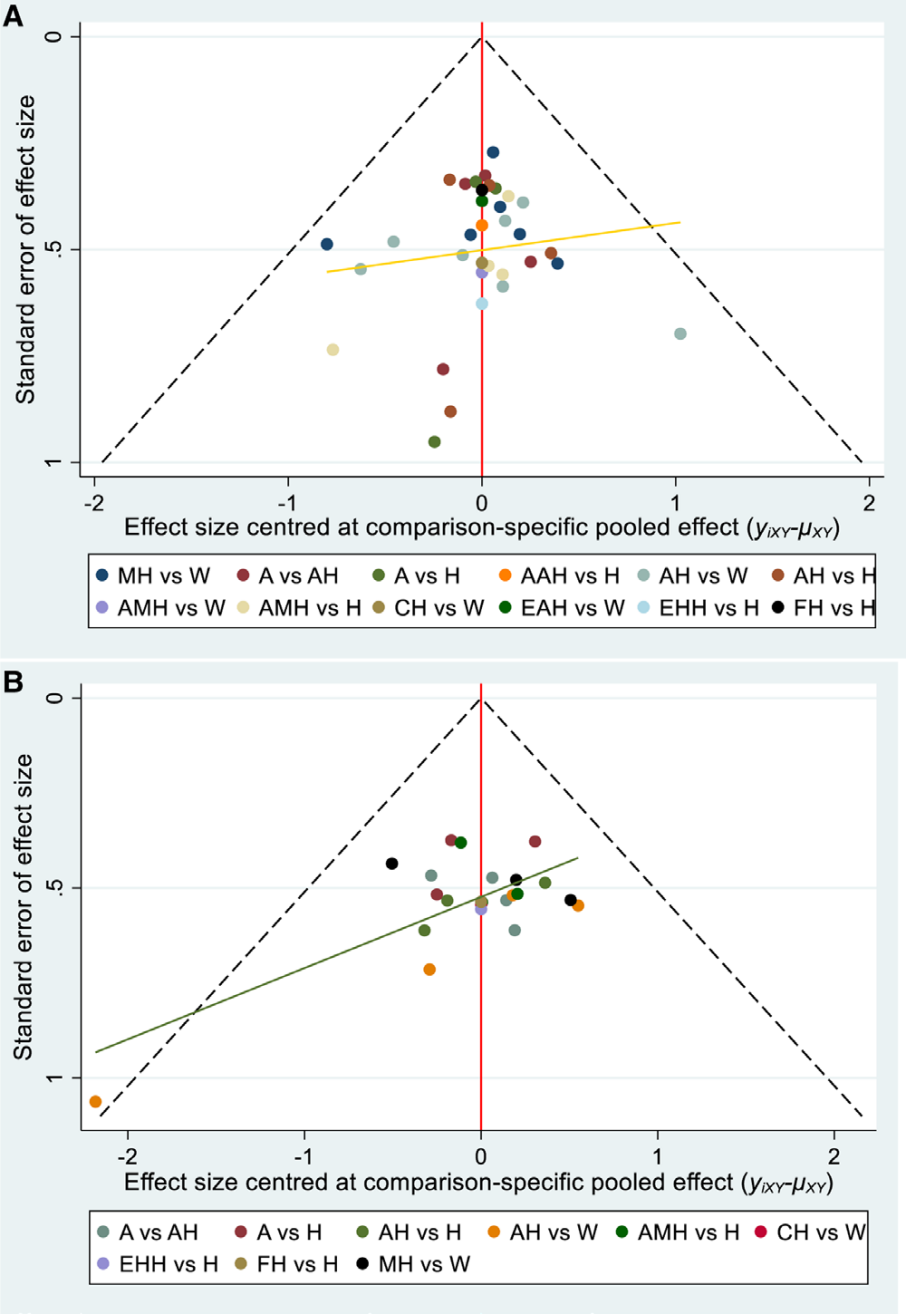
The funnel plot of included studies.

In the comparison-corrected funnel plot, the dots in the funnel chart of different colors represent direct comparisons between different therapies, with the number of dots indicating the number of studies. Regarding the pregnancy rate outcome indicators, which consist of 12 distinct comparison groups for the interventions, most of the dots in the funnel plot were symmetrically distributed on the vertical line and its corresponding sides. However, the scatterplot of the points indicates the possibility of some degree of publication bias (Fig. 8A). Regarding the ovulation rate outcome indicators, which comprise 8 distinct comparison groups for the interventions, most of the dots in the funnel plot were symmetrically distributed on the vertical line and its corresponding sides. However, one group stands out as being further away from the regression line, suggesting the presence of some degree of publication bias (Fig. 8B).

## 4. Discussion

### 4.1. Results analysis

The findings of this study demonstrate that moxibustion + herbal, fire acupuncture + herbal, acupuncture + herbal, electroacupuncture + herbal, and acupoint application + herbal have some degree of advantages when exhibited improved the clinical pregnancy rate among PCOS-related infertility in comparison to monotherapy involving acupuncture, herbal medicine, and western medicine. Moreover, ear point pressure + herbal Enema + herbal, acupuncture and moxibustion + herbal, fire acupuncture + herbal, and acupuncture + Chinese herbal medicine also have some degree of advantages when enhanced ovulation rate in PCOS-related infertility compared to monotherapy comprising of acupuncture, herbal, and western medicine. When combined TCM therapy was used as alternative therapies, the cumulative probability ranking suggested that the top 3 efficacies in terms of improving pregnancy rate were moxibustion + herbal, fire acupuncture + herbal, and acupuncture + herbal, and the top 3 efficacies in terms of improving ovulation rate were fire acupuncture + herbal, acupuncture + herbal, ear point pressure + herbal enema + herbal. In summary, it is evident that the optimal treatment programs varied based on the outcome indicators, and there was no optimal treatment program for each outcome indicator.

In the TCM, there is no specific term for PCOS, but based on its clinical symptoms, it can be classified as “amenorrhea,” “lower abdominal masses,” “infertility,” “scanty menstruation,” and so on. Due to the complexity of PCOS, Chinese medicine experts have varying opinions on its etiology and pathomechanism, but generally agree that it can be treated through regulating the functions of the kidney, spleen, and liver. Previous studies, when statistically analyzing the TCM syndromes of PCOS, showed that kidney deficiency is the main syndrome of PCOS and has the highest frequency of occurrence.^[53]^ On one hand, kidney essence insufficiency can lead to the inability to nourish the *tiangui* and affect the development and maturation of the ovum. On the other hand, kidney essence insufficiency can involve the functions of the liver and spleen, generating phlegm-dampness and blood stasis, hinder the function of the *Chongren,* uterus or skin, causing difficulty with the discharge of ovums and symptoms such as hirsutism, obesity, and acne. It can be seen that this disease is based on kidney deficiency, involving the liver and spleen, phlegm-dampness and blood stasis as the target, interacting with each other, disrupting the dynamic balance of the reproductive axis of “kidney – *tiangui* – *Chongren*—uterus” and causing metabolic dysfunction of the body, thus affecting women’s pregnancy and impregnation in addition morbidity.^[77]^

As an adjunctive therapy, TCM can offer personalized treatment from a holistic perspective combining pattern differentiation and treatment. Recent research has indicated that TCM effectively manages PCOS through herbal formulations and acupuncture.^[78]^ Modern pharmacological examination has validated the efficacy of many herbal monomers. A series of herbals that tonify the kidney, fortify the spleen, dissolve phlegm, dry dampness, invigorate blood and dissolve stasis, can regulate hormone levels, metabolic indexes, and localized ovarian perfusion, which enhance the ovarian and uterine microenvironment, thicken endometrium, promote follicular growth and follicle expulsion, and ultimately improve pregnancy outcomes and relieve other symptoms.^[79–82]^ The most common cycle-regulating therapy in TCM can regulate menstruation and promote ovulation through the patterns of *qi* and blood and *yin-yang* changes and the administration of medication at different stages.^[83]^ Its mechanism is being gradually explored. Xu et al^[84]^ discovered that adjusting the weekly cycle method can improve local microcirculation in PCOS patients, increasing blood supply to the uterus and ovaries, promoting follicle development, and ovulation. Shen et al revealed that this method can also reduce serum levels of LH, LH/FSH, hirsuteness, and acne incidence while improving ovulation rate.^[85]^ Acupuncture treatment can support the body, improve kidney function, strengthen the spleen, and regulate *qi* and blood flow by selecting appropriate acupoint pairings, such as Sanyinjiao (SP6), Guanyuan (RN4), Zigong (EX-CA1), Zhongji (RN3), and Qihai (RN6).^[86]^ Additionally, acupuncture can be tailored to address specific symptoms by adding or subtracting acupressure points. Biologically, acupuncture can regulate hypothalamic function, pituitary neurotransmitter release, and ovarian function, which in turn adjusts hormone levels and the uterine microenvironment.^[87,88]^ Animal experiments have illustrated that electroacupuncture can stimulate homeostasis of the hypothalamic–pituitary–ovarian axis in normal female rats and regulate levels of gonadotropin-releasing hormone, luteinizing hormone, follicle-stimulating hormone, estradiol, and progesterone.^[89]^ Furthermore, acupuncture may improve follicular maturation in patients with PCOS by modulating ovarian innervation and decreasing elevated levels of nerve growth factor in the ovaries, in order to achieve the desired therapeutic effect.^[88,90]^

In summary, TCM has shown significant efficacy in treating PCOS. Although there are numerous therapies with significant efficacy, the adverse effects of the drugs limit their clinical applicability. Specific molecular studies and toxicology tests of Chinese herbs are still lacking and require further clarification to provide effective targeted therapy for PCOS. Nonetheless, the interventions mentioned above provide some reference for clinical application and research. Please note that the probability ranking results are for reference purposes only.

### 4.2. Strengths and limitations of this study

*Strengths*: The literature screening was focused on minimizing differences by focusing on diagnostic, inclusion, exclusion, and efficacy criteria of the included studies. This study selected commonly used TCM therapies to treat PCOS infertility, which expands the range of treatment options, compares interventions, and explores targeted and efficient therapies.

*Limitations*: The number of outcome indicators in this paper is limited, and the number of RCTs included under different interventions is small. The overall quality of the RCTs included in this paper is not high, and most of the literature did not use allocation concealment methods and record case shedding, which may impact the evaluation of results. The types of Chinese medicine symptoms, the start time of the interventions, and the intervention course of the included studies are inconsistent, which may result in differences in outcome measurements. The included studies examined different types of Chinese medicine symptoms about PCOS infertility, and subgroup analyses could not be performed due to limitations in the literature.

### 4.3. Implications for future research

#### 4.3.1. Trial design

A majority of the studies included had small sample sizes, and there was limited utilization of blinding methods with concealed allocation. Blinding is a critical component in reducing potential biases in results, especially for subjectively measured outcomes. While achieving complete double-blinding or triple-blinding designs in acupuncture studies may be challenging, blinding of acupuncture subjects or data collectors or evaluators can help minimize subjective factors and result biases. Additionally, partially randomized patient preference clinical trials may be an effective approach to address these limitations.^[91,92]^

#### 4.3.2. Adverse reactions

Adverse events play a critical role in clinical trials as they provide important safety information for evaluation. To ensure objective and accurate data collection, future studies should document adverse events in accordance with the clinical protocol.

#### 4.3.3. In terms of diagnostic and efficacy criteria

The included studies lacked uniform diagnostic reference criteria. In future research, it would be beneficial to establish validated diagnostic criteria based on existing literature or guideline recommendations to enhance measurement accuracy and promote comparability with other studies in the field.

In conclusion, future research should adhere to the requirements of controlled trials, rigorously control diagnostic criteria, reference previous studies or guideline outcome indicators for assessment, design and implement large-scale trials, and faithfully record adverse reactions and safety assessments to achieve higher-quality RCTs.

## 5. Conclusions

This research has demonstrated that the combined TCM therapy was effective in improving clinical pregnancy and ovulation rate when used as an alternative treatment for PCOS infertility. Moxibustion combined with herbal may become the optimal therapy to improve pregnancy rate, and fire acupuncture combined with herbal may become the optimal therapy to improve ovulation rate, which has some clinical reference value. In clinical application, the results of this study can be appropriately referred to and appropriate therapies can be selected according to the specific conditions of patients. However, it is important to acknowledge that further research is necessary to verify these findings via large samples RCTs that are of high quality and standardized in nature.

## Author contributions

**Conceptualization:** Yun-Peng Deng, Yan-Li Zhou, Xu-Guang Yang.

**Data curation:** Yun-Peng Deng, Xu-Guang Yang.

**Investigation:** Yan-Li Zhou, Teng-Teng Wei, Guang-Shuai He, Zhi-Xin Zhu, Shu-Ning Zhang, Mei-Jun Liu, Jing-Jing Xue, Wei-Xing Zhang.

**Methodology:** Yun-Peng Deng, Xu-Guang Yang.

**Software:** Yun-Peng Deng, Teng-Teng Wei, Guang-Shuai He, Zhi-Xin Zhu, Shu-Ning Zhang, Mei-Jun Liu, Jing-Jing Xue, Wei-Xing Zhang.

**Validation:** Teng-Teng Wei, Guang-Shuai He, Zhi-Xin Zhu, Shu-Ning Zhang, Mei-Jun Liu, Jing-Jing Xue, Wei-Xing Zhang.

**Visualization:** Yan-Li Zhou.

**Writing – original draft:** Yun-Peng Deng.

**Writing – review & editing:** Yun-Peng Deng, Xu-Guang Yang.

## References

[1] Azziz R. Polycystic ovary syndrome. Obstet Gynecol. 2018;132:321–36. 29995717 10.1097/AOG.0000000000002698

[2] Bozdag G Mumusoglu S Zengin D Karabulut E Yildiz BO. The prevalence and phenotypic features of polycystic ovary syndrome: a systematic review and meta-analysis. Hum Reprod. 2016;31:2841–55. 27664216 10.1093/humrep/dew218

[3] Jin J Ruan X Hua L. Prevalence of diminished ovarian reserve in Chinese women with polycystic ovary syndrome and sensitive diagnostic parameters. Gynecol Endocrinol. 2017;33:694–7. 28412857 10.1080/09513590.2017.1310838

[4] Collée J Mawet M Tebache L Nisolle M Brichant G. Polycystic ovarian syndrome and infertility: overview and insights of the putative treatments. Gynecol Endocrinol. 2021;37:869–74. 34338572 10.1080/09513590.2021.1958310

[5] Sadeghi HM Adeli I Calina D. Polycystic ovary syndrome: a comprehensive review of pathogenesis, management, and drug repurposing. Int J Mol Sci. 2022;23:583. 35054768 10.3390/ijms23020583 PMC8775814

[6] Li W Chen Q Xie Y Hu J Yang S Lin M. Prevalence and degree of insulin resistance in Chinese Han women with PCOS: results from euglycemic-hyperinsulinemic clamps. Clin Endocrinol (Oxf). 2019;90:138–44. 30229990 10.1111/cen.13860 PMC7380049

[7] Thakker D Raval A Patel I Walia R. N-acetylcysteine for polycystic ovary syndrome: a systematic review and meta-analysis of randomized controlled clinical trials. Obstet Gynecol Int. 2015;2015:817849. 25653680 10.1155/2015/817849 PMC4306416

[8] Practice Committee of the American Society for Reproductive Medicine. Electronic address: asrm@asrm.org. Definitions of infertility and recurrent pregnancy loss: a committee opinion. Fertil Steril. 2020;113:533–5. 32115183 10.1016/j.fertnstert.2019.11.025

[9] Vander Borght M Wyns C. Fertility and infertility: definition and epidemiology. Clin Biochem. 2018;62:2–10. 29555319 10.1016/j.clinbiochem.2018.03.012

[10] Fu B Qin N Cheng L. Development and validation of an infertility Stigma Scale for Chinese women. J Psychosom Res. 2015;79:69–75. 25499618 10.1016/j.jpsychores.2014.11.014

[11] Expert Group of Consensus on Infertility Management & Fertility Preservation Related to Polycystic Ovary Syndrome; Reproductive Endocrinology & Fertility Preservation Section of Chinese Society on Fertility Preservation under Chinese Preventive Medicine Association. Consensus on infertility management and fertility preservation related with polycystic ovary syndrome. J Reprod Med. 2020;29:843–51.

[12] Ong M Peng J Jin X Qu X. Chinese herbal medicine for the optimal management of polycystic ovary syndrome. Am J Chin Med. 2017;45:405–22. 28359195 10.1142/S0192415X17500252

[13] Domecq JP Prutsky G Mullan RJ. Adverse effects of the common treatments for polycystic ovary syndrome: a systematic review and meta-analysis. J Clin Endocrinol Metab. 2013;98:4646–54. 24092830 10.1210/jc.2013-2374 PMC5399491

[14] Sun ML Bai WP Song QK. Metformin with or without clomiphene citrate versus laparoscopic ovarian drilling with or without clomiphene citrate to treat patients with clomiphene citrate-resistant polycystic ovary syndrome: a systematic review and meta-analysis. Front Pharmacol. 2022;13:576458. 35814214 10.3389/fphar.2022.576458 PMC9256960

[15] Armanini D Boscaro M Bordin L Sabbadin C. Controversies in the pathogenesis, diagnosis and treatment of PCOS: focus on insulin resistance, inflammation, and hyperandrogenism. Int J Mol Sci. 2022;23:4110. 35456928 10.3390/ijms23084110 PMC9030414

[16] Escobar-Morreale HF. Polycystic ovary syndrome: definition, aetiology, diagnosis and treatment. Nat Rev Endocrinol. 2018;14:270–84. 29569621 10.1038/nrendo.2018.24

[17] Jiahui W Zengxiang M. Research progress on direct economic burden of polycystic ovary syndrome and its complications. Health Econ Res. 2022;39:24–7.

[18] Shunli H Qitian L Bingyi Y Weiwei Z Xiuqi Y. Ziyu decoction in treatment of infertility with polycystic ovary syndrome of kidney deficiency. ActaChinese Med. 2023;38:874–8.

[19] Shuguang Z Hang Z Yang Y Qiaozhi Y Linwen D. Progress on hot spot analysis and development trend of traditional Chinese medicine treatment mechanism of polycystic ovary syndrome research on visual spectrogram. J Chengdu Univ Tradit Chin Med. 2022;45:92–7.

[20] Yang H Xiao ZY Yin ZH. Efficacy and safety of acupuncture for polycystic ovary syndrome: an overview of systematic reviews. J Integr Med. 2023;21:136–48. 36635165 10.1016/j.joim.2022.12.002

[21] Wu J Chen D Liu N. Effectiveness of acupuncture in polycystic ovary syndrome: a systematic review and meta-analysis of randomized controlled trials. Medicine (Baltim). 2020;99:e20441. 10.1097/MD.0000000000020441 PMC12245336 32481448

[22] Yanjuan S Fengxia L Song W. Network meta-analysis on the effects of the acupuncture-related therapy on ovulation rate and pregnancy rate in patients with polycystic ovary syndrome. Chin Acupunct Moxibustion. 2019;39:792–8. 10.13703/j.0255-2930.2019.07.029 31286745

[23] Shouqiang H Haiyan X Jun X Jie X Fanghui H. Efficacy of acupuncture for PCOS infertility: a systematic review. Chin J Evid-Based Med. 2021;21:431–7.

[24] Yue Y Tong C Zheng Z Xinmin L. Efficacy and safety of oral Chinese patent medicines in treatment of polycystic ovary syndrome: a network Meta-analysis. Chin Tradit Herb Drugs. 2022;53:7477–90.

[25] Yun L Liqun W Shuqi Y Chunxiao W Liming L Wei Y. Acupuncture for infertile women without undergoing assisted reproductive techniques (ART): a systematic review and meta-analysis. Medicine (Baltim). 2019;98:e16463. 10.1097/MD.0000000000016463 PMC6709164 31335705

[26] Zhu P Guan JZ Hai QC Jin J Shi L Hua L. The clinical effectiveness and safety of traditional Chinese medicine Jinfeng pill in adjuvant treatment of infertility with polycystic ovary syndrome: a protocol for systematic review and meta-analysis. Medicine (Baltim). 2022;101:e28676. 10.1097/MD.0000000000028676 PMC8797503 35089214

[27] Lin Y Xiang L Li X Tang Q Meng F Chen W. Exploring the mechanism of Yi-Jing decoction in treating polycystic ovary syndrome by using network pharmacology. Curr Med Chem. 2023;30:2463–74. 35532255 10.2174/0929867329666220508180611

[28] Kun M Linjuan G Yanxia C Caidie T. Study on mechanism of Bushen Culuan formula in treatment of polycystic ovary syndrome based on network pharmacology and molecular docking. China J Chin Materia Medica. 2021;46:2650–9. 10.19540/j.cnki.cjcmm.20210312.501 34296561

[29] Pan X Liu Y Liu L. Bushen Jieyu Tiaochong formula reduces apoptosis of granulosa cells via the PERK-ATF4-CHOP signaling pathway in a rat model of polycystic ovary syndrome with chronic stress. J Ethnopharmacol. 2022;292:114923. 34923086 10.1016/j.jep.2021.114923

[30] Mingsan M Mengfan P Xiaoli Y. Effect of dodder total flavone on polycystic ovary syndrome rat models. Chin J Exp Tradit Med Formulae. 2019;25:143–50.

[31] Wang W Zheng J Cui N. Baicalin ameliorates polycystic ovary syndrome through AMP-activated protein kinase. J Ovarian Res. 2019;12:109. 31722718 10.1186/s13048-019-0585-2 PMC6852906

[32] Yan Q Luo K Tang R. Research progress of traditional Chinese medicine therapy on PCOS. Acad J Med Health Sci. 2023;4:84–92.

[33] Lumley T. Network meta-analysis for indirect treatment comparisons. Stat Med. 2002;21:2313–24. 12210616 10.1002/sim.1201

[34] Rouse B Chaimani A Li T. Network meta-analysis: an introduction for clinicians. Intern Emerg Med. 2017;12:103–11. 27913917 10.1007/s11739-016-1583-7 PMC5247317

[35] Watt J Tricco AC Straus S Veroniki AA Naglie G Drucker AM. Research techniques made simple: network meta-analysis. J Invest Dermatol. 2019;139:4–12.e1. 30579427 10.1016/j.jid.2018.10.028

[36] Zhang X Cheng B Zhang Y. A hands-on tutorial for systematic review and meta-analysis with example data set and codes. J Speech Lang Hear Res. 2022;65:3217–38. 36001816 10.1044/2022_JSLHR-21-00607

[37] Phillips MR Steel DH Wykoff CC. A clinician’s guide to network meta-analysis. Eye (Lond). 2022;36:1523–6. 35145277 10.1038/s41433-022-01943-5 PMC9307840

[38] Liang P Li Y Feng Y. Effects of acupuncture-related therapies in the rehabilitation of patients with post-stroke aphasia-a network meta-analysis of randomized controlled trials. Brain Sci. 2022;12:1282. 36291216 10.3390/brainsci12101282 PMC9599621

[39] Rotterdam ESHRE/ASRM-Sponsored PCOS consensus workshop group. Revised 2003 consensus on diagnostic criteria and long-term health risks related to polycystic ovary syndrome (PCOS). Hum Reprod. 2004;19:41–7. 14688154 10.1093/humrep/deh098

[40] Editor-in-Chief, Nanchong Medical College. Obstetrics and Gynecology. (妇产科学). Bei Jing: People’s Health Publishing House. 1981;07:245–8.

[41] Xiaoling F Tingting Z. Gynecology of Chinese Medicine. (中医妇科学) Version 5. Bei Jing: China Chinese Medicine Publishing House. 2021;08:1–368.

[42] Sterne JAC Savović J Page MJ. RoB 2: a revised tool for assessing risk of bias in randomised trials. BMJ. 2019;366:l4898. 31462531 10.1136/bmj.l4898

[43] Jackson D Riley R White IR. Multivariate meta-analysis: potential and promise. Stat Med. 2011;30:2481–98. 21268052 10.1002/sim.4172 PMC3470931

[44] Lun L Jinhui T. Mesh Meta-analysis Methodology and Practice. (网状Meta分析方法与实践). Beijing: China Medical Science and Technology Press. 2017:73.

[45] Hutton B Salanti G Caldwell DM. The PRISMA extension statement for reporting of systematic reviews incorporating network meta-analyses of health care interventions: checklist and explanations. Ann Intern Med. 2015;162:777–84. 26030634 10.7326/M14-2385

[46] Zhang D Wu J Liu S Zhang X Zhang B. Network meta-analysis of Chinese herbal injections combined with the chemotherapy for the treatment of pancreatic cancer. Medicine (Baltim). 2017;96:e7005. 10.1097/MD.0000000000007005 PMC5457895 28538415

[47] Guyot P Ades AE Ouwens MJ Welton NJ. Enhanced secondary analysis of survival data: reconstructing the data from published Kaplan-Meier survival curves. BMC Med Res Methodol. 2012;12:9. 22297116 10.1186/1471-2288-12-9 PMC3313891

[48] Chaimani A Higgins JP Mavridis D Spyridonos P Salanti G. Graphical tools for network meta-analysis in STATA. PLoS One. 2013;8:e76654. 24098547 10.1371/journal.pone.0076654 PMC3789683

[49] Cuimin J. Clinical observation on treating 60 cases of spleen deficiency type PCOS infertility in TCM and moxibustion. Clin J Chin Med. 2012;4:47–8.

[50] Yugang X. Observation on therapeutic effect of traditional Chinese medicine and moxibustion in treating 128 cases of Spleen kidney yang deficiency type polycystic ovary syndrome induced infertility. World Chin Med. 2014;9:1079–82.

[51] Lijuan Z JuLan H ShaoFang X. The clinical observation of Cang fu Daotan decoction combined with acupoint heat － sensitive moxibustion on infertility caused by phlegm － dampness internal obstruction polycystic ovary syndrome. J Jiangxi Univ Chin Med. 2019;31:58–60.

[52] Qing S. Observation of clinical effects of Chinese herbal medicine plus moxibustion in the treatment of polycystic ovary syndrome infertility of spleen-kidney yang deficiency type. (观察中药加艾灸治疗脾肾阳虚型多囊卵巢综合症不孕的临床效果). World Latest Med Inf. 2016;16:93–4.

[53] Jun L Jian L Yuting H. Clinical study on heat-sensitive moxibustion combined with traditional Chinese medicine for Bushen Huoxue Huatan to treat obesity PCOS infertility. Med Innov China. 2019;16:52–6.

[54] Zhifang L Haiqiong C. Effect of self-developed formula for warming the kidney and strengthening the spleen combined with acupuncture point moxibustion on Infertility patients with polycystic ovary syndrome with spleen-kidney yang deficiency. (自拟温肾健脾方结合穴位艾灸对脾肾阳虚型多囊卵巢综合征不孕症患者的影响). Women’s Health Res. 2017;16:153–4.

[55] Yanli P Yan S Lan L Yanbing H. Effect of Bushen Huoxue recipe combined with electroacupuncture on endocrine hormone and pregnancy outcome in infertile patients with polycystic ovary syndrome. Guangxi Med J. 2018;40:795–8.

[56] Hongqiu Z Jiaojie L Lu Z Ting C Jiaojiao H. Clinical analysis of triple therapy of TCM on ovulation induction in infertile patients with kidney － deficiency and liver － depression type of polycystic ovary syndrome. Hebei J Tradit Chin Med. 2016;38:360–363 + 372.

[57] Yining G. Effects of fire-needle therapy combined with Zuo Gui Shu Gan decoction on ovulation and serum testosterone level in infertile patients with polycystic ovary syndrome. Shanghai J Acu-mox. 2020;39:406–10.

[58] LiJuan Z Julan H Shaofang X. Clinical study of Cangfu Daotan decoction combined with acupoint catgut embedding in the treatment of infertility of polycystic ovary syndrome with phlegm dampness and internal obstruction. J Jiangxi Univ Chin Med. 2020;32:68–70.

[59] Ying S. Application of the combination of Yi kidney and blood stasis formula with acupuncture point application in the treatment of infertility in polycystic ovary syndrome with kidney deficiency and blood stasis. (益肾化瘀方联合穴位贴敷在肾虚血瘀型多囊卵巢综合征不孕治疗中的应用). Inner Mongolia Med J. 2022;54:985–7.

[60] Linchong Z Yu Y. Clinical curative effect of acupuncture combined with Bushen Jieyu decoction on infertility of polycystic ovary syndrome of liver-stagnation kidney deficiency type and its influences on ovarian function. J Hubei Univ Chin Med. 2022;24:95–8.

[61] Fenglan R Lianjie Y Yuchun L. Efficacy observation of acupuncture combined with medication for infertility in polycystic ovary syndrome of kidney deficiency and liver depression pattern. Shanghai J Acu-mox. 2022;41:43–9.

[62] Na L Feng-Lan R. Observations on the efficacy of acupuncture plus Zuogui Wenjing decoction for infertility due to polycystic ovarian syndrome. Shanghai J Acu-mox. 2021;40:1346–52.

[63] Hongwei Y Xuejuan Z Meixia W Xiuming L. Clinical study on Yishen Huatan prescription combined with acupuncture for infertility due to polycystic ovary syndrome of kidney deficiency with phlegm-dampness type. New Chin Med. 2022;54:153–7.

[64] Yuting H Jun L. Clinical observation on alternate acupuncture combined with regulating the menstrual cycle method in the treatment of polycystic ovary syndrome infertility of kidney deficiency and phlegm dampness type. Chin Med Mod Distance Educ China. 2021;19:96–8.

[65] Duosheng J Yingchun Z Xianqun W Song W. Infertility in polycystic ovary syndrome treat with acupuncture and clomiphene: a randomized controlled trial. Chin Acupunct Moxibustion. 2015;35:114–8. 25854013

[66] Qiuzhu Z. Observations on the efficacy of acupuncture and moxibustion plus menstrual cycle-regulating method for infertility in patients with polycystic ovary syndrome of spleen deficiency and phlegm-dampness type. Shanghai J Acu-mox. 2019;38:1253–7.

[67] Huimin P. Clinical study on the combination of acupuncture and medicine in the treatment of polycystic ovary syndrome infertility with evidence of kidney deficiency and phlegm-dampness. (针药结合治疗多囊卵巢综合征不孕症肾虚痰湿证临床研究). J Pract Tradit Chin Med. 2022;38:1290–2.

[68] Yan C. Clinical research of regulating menstrual circsdian rhythm method combined with acupuncture to treat infertile women with polycystic ovary syndrome cased by kidney deficiency and phlegmatic hygrosis. (调周法结合针灸治疗肾虚痰湿型PCOS不孕患者的临床研究) [dissertation]. Nanjing Univ Chin Med. 2015.

[69] Shanxing Q. The clinical research on Chinese medicine sequencing with the Acupuncture treat anovulation infertility caused by PCOS. (中药序贯配合针灸治疗PCOS致排卵障碍性不孕症的临床研究). Nanjing Univ Chin Med. 2012.

[70] Yajiao W. 56 cases of obese polycystic ovary syndrome infertility treated with acupuncture and traditional Chinese medicine. (针刺配合中药治疗肥胖型多囊卵巢综合征不孕症56例). Fujian J Tradit Chin Med. 2013;44:6–7 + 9.

[71] Yi S. Efficacy of acupuncture combined with traditional Chinese medicine and letrozole in the treatment of polycystic ovary syndrome infertility (kidney deficiency, phlegm and dampness type). (针灸联合中药与来曲唑治疗多囊卵巢综合征不孕症(肾虚痰湿型)的疗效观察). Electronic J Pract Gynecol Endocrinol. 2017;4:98 + 100.

[72] Ran L Pengxuan Y Xiaotong Z Weijun Z Lu Z Jianwu S. Effect of acupuncture and medicine on infertility due to polycystic ovary syndrome with kidney deficiency. China Med Herald. 2018;15:149–52.

[73] Xiaojing L Hao Z Zhiling Z Beining G. Clinical observation of Zuogui drink and Huanglian Ejiao decoction combined with acupuncture in treatment of infertility associated with polycystic ovary syndrome. J Guangzhou Univ Tradit Chin Med. 2021;38:2145–51.

[74] Linling Y. Evaluation of the effect of self-formulated menstrual regulation soup combined with acupuncture in the treatment of polycystic ovary syndrome infertility. (自拟调经汤结合针刺治疗多囊卵巢综合征性不孕症的效果评价). Health Required. 2020;22:225.

[75] Shumin G Xiupeng R Yeting W Lijie Z Wentao M Xuejing C. Clinical study on the treatment of phlegm-damp polycystic ovary syndrome combined with infertility by Chinese herbal medicine. (中医中药治疗痰湿型多囊卵巢综合征合并不孕症临床研究). Diet Health. 2017;4:98–9.

[76] Chengwen L. Combination of acupuncture and medicine in the treatment of infertility in patients with polycystic ovary syndrome. (针药结合治疗多囊卵巢综合征患者不孕不育的效果). World Clin Med. 2019;13:203,206.

[77] Ye W Deng G Yin L Ye J. Efficacy and safety of moxibustion in the treatment of infertility with polycystic ovary syndrome: a protocol of systematic review and meta-analysis. Medicine (Baltim). 2021;100:e24529. 10.1097/MD.0000000000024529 PMC7982196 33725936

[78] Jing L Zimeng P Chang S. Research progress on mechanism of oxidative stress in polycystic ovary syndrome and treatment of traditional Chinese medicine. Shanghai J Tradit Chin Med. 2022;56:96–102.

[79] Yilei Z Wen P Yongjuan W. Progress of Chinese and Western medicine research on the etiology and pathogenesis of polycystic ovary syndrome. (多囊卵巢综合征病因病机中西医研究进展). J Basic Chin Med. 2016;22:1004–6.

[80] Yan L Yongge G Yue L Wanying F Yang S. Systematic review on the effect of Chinese herbal medicine combined with health guidance on polycystic ovary syndrome on the basis of theory of preventive treatment of disease. Modernization Tradit Chin Med Materia Medica-World Sci Technol. 2022;24:4473–83.

[81] Bin Y Cancan H Quansheng W. Effect of Yi kidney pro-ovulation formula combined with letrozole on ovulation and ovarian blood perfusion in luteinized infertile patients with unruptured follicles. (益肾促排方联合来曲唑对未破裂卵泡黄素化不孕患者排卵及卵巢血流灌注的影响). Lishizhen Med Materia Medica Res. 2023;34:122–4.

[82] Moini Jazani A Nasimi Doost Azgomi H Nasimi Doost Azgomi A Nasimi Doost Azgomi R. A comprehensive review of clinical studies with herbal medicine on polycystic ovary syndrome (PCOS). Daru. 2019;27:863–77. 31741280 10.1007/s40199-019-00312-0 PMC6895349

[83] Lixue F Shicong Q Yan L Lihui H. Traditional Chinese medicine in treatment of polycystic ovary syndrome. Chin Arch Tradit Chin Med. 2017;35:166–8.

[84] Huayun X Jinrong F. Effects of CAI Xiaosun’s menstruation periodic therapy on hemodynamics of uterine artery and ovarian artery in infertility patients with polycystic ovary syndrome. J Tradit Chin Med. 2014;55:129–32.

[85] Wenjuan S Tianjiao Y Bao J. Advances in etiology pathogenesis and treatment of polycystic ovary syndrome. Liaoning J Tradit Chin Med. 2021;48:196–9.

[86] Chuanlan J Ran P Lipeng X Zhongchao W Jiping Z. Clinical rules for acupoint selection and prescription composition in treatment of polycystic ovary syndrome with acupuncture. Chin Acupunct Moxibustion. 2015;35:625–30. 26480575

[87] Shuang L Meiling Y Jiulong W Jiulong W Shengfeng L. Analyzing the feasibility of acupuncture intervention for ovulation obstructive infertility. Chin J Integr Tradit West Med. 2017;37:870–4.

[88] Yi S Jing W. Acupuncture modulates the hypothalamic-pituitary-ovarian axis in the treatment of polycystic ovary syndrome: a research progress. Chin J Integr Tradit West Med. 2022;42:625–32.

[89] Tong X Liu Y Xu X. Ovarian innervation coupling with vascularity: the role of electro-acupuncture in follicular maturation in a rat model of polycystic ovary syndrome. Front Physiol. 2020;11:474. 32547407 10.3389/fphys.2020.00474 PMC7273926

[90] Zhu H Nan S Suo C. Electro-acupuncture affects the activity of the hypothalamic-pituitary-ovary axis in female rats. Front Physiol. 2019;10:466. 31068836 10.3389/fphys.2019.00466 PMC6491808

[91] Yiying W Jianping L Huijuan C. Application cases analysis of PRPP design in evaluation of curative effect of non-drug therapy of traditional Chinese medicine. Beijing J Tradit Chin Med. 2021;40:546–8.

[92] Wasmann KA Wijsman P van Dieren S Bemelman W Buskens C. Partially randomised patient preference trials as an alternative design to randomised controlled trials: systematic review and meta-analyses. BMJ Open. 2019;9:e031151. 10.1136/bmjopen-2019-031151 PMC6797441 31619428

